# Enhancement of the Plant Grafting Technique with Dielectric Barrier Discharge Cold Atmospheric Plasma and Plasma-Treated Solution

**DOI:** 10.3390/plants11101373

**Published:** 2022-05-22

**Authors:** Evgeny M. Konchekov, Leonid V. Kolik, Yury K. Danilejko, Sergey V. Belov, Konstantin V. Artem’ev, Maxim E. Astashev, Tatiana I. Pavlik, Vladimir I. Lukanin, Alexey I. Kutyrev, Igor G. Smirnov, Sergey V. Gudkov

**Affiliations:** 1 Prokhorov General Physics Institute of the Russian Academy of Sciences, 119991 Moscow, Russia; leonidkolik@mail.ru (L.V.K.); dyuk42@list.ru (Y.K.D.); ser79841825@yandex.ru (S.V.B.); artemievkv@mail.ru (K.V.A.); astashev.max@gmail.com (M.E.A.); ti.pavlik.u@gmail.com (T.I.P.); vladimirlukanin@yandex.ru (V.I.L.); s_makariy@rambler.ru (S.V.G.); 2 Federal Scientific Agroengineering Center VIM, 109428 Moscow, Russia; alexeykutyrev@gmail.com (A.I.K.); rashn-smirnov@yandex.ru (I.G.S.)

**Keywords:** pear, plasma-activated water, *Pyrus communis* L., reactive nitrogen species, reactive oxygen species

## Abstract

A garden plant grafting technique enhanced by cold plasma (CAP) and plasma-treated solutions (PTS) is described for the first time. It has been shown that CAP created by a dielectric barrier discharge (DBD) and PTS makes it possible to increase the growth of *Pyrus communis* L. by 35–44%, and the diameter of the root collar by 10–28%. In this case, the electrical resistivity of the graft decreased by 20–48%, which indicated the formation of a more developed vascular system at the rootstock–scion interface. The characteristics of DBD CAP and PTS are described in detail.

## 1. Introduction

The main task in nursery is to obtain high-quality planting material in a minimum period of time. To do this, it is necessary to optimize the process of graft assemblage between the rootstock and scion. The following factors can be the reasons for poor assembly: differences in structure, different growth rates of the graft components, different cambium activity, changes caused by viruses, etc. The search and application of physical methods that improve the quality of survival of rootstock–scion combinations remain relevant [1]. To solve this problem, the use of low-temperature plasma seems promising; it is widely used in areas of natural sciences such as chemistry, physics, biology, medicine, and agriculture. Since the 1980s, when low-temperature plasma was used to inactivate bacteria, a large number of original articles have appeared in the literature describing new applications of low-temperature plasma in the life sciences. Most of these works are summarized in specialized review articles [2,3,4,5,6]. In addition to phenomenology, numerous articles have been devoted to the mechanisms of interaction of plasma with the organism, including practical applications in medicine, veterinary medicine, and plant growing [7,8,9,10,11,12,13,14,15,16,17]. In such applications, a non-equilibrium low-temperature plasma of atmospheric pressure is used, in which the temperature of the gaseous medium (rotational temperature of ions) adjacent to the biological object does not exceed 40 °C. This plasma is called cold atmospheric plasma (CAP). Studies have shown that the biological effects of CAP are mainly due to the action of reactive oxygen species (ROS) and reactive nitrogen species (RNS). The nature of plasma-chemical reactions under the influence of CAP is rather complex. The main reagents are superoxide radical, hydroxyl radical, hydroperoxyl radical, singlet oxygen, nitric oxide, and peroxynitrite [18,19]. The lifetime of most of these compounds is short, for example, the lifetime of the hydroxyl radical is of the order of 1 ns, and the lifetime of the superoxide radical is of the order of 10 μs. These compounds can be registered only at the moment of interaction of the plasma with the substance. Typically, the interaction model of plasma with biological tissues is interaction with aqueous solutions. This is due to two reasons; firstly, biological objects consist mainly of such solutions, and secondly, most of the chemical reactions initiated by CAP occur in the liquid and at the air–liquid interface. As a result of exposure, there is a change in physicochemical characteristics such as pH, redox potential, and electrical conductivity. Long-lived compounds are formed in liquids: hydrogen peroxide, ozone, and nitrogen oxides (NOxs) [20,21]. Treatment of biological objects with plasma-treated solutions (PTSs) is also a promising approach that allows to achieve a similar result compared to the direct action of CAP [22,23,24,25,26]. Currently, there are many methods for CAP and PTS generation: based on a dielectric barrier discharge or other types of discharges, using noble gases, or operating in atmospheric air [27,28,29,30,31,32]. Despite the widespread use of CAP in the woodworking industry [32], there is no information in the literature on the use of CAP in plant grafting. In this work, we are the first to study the effect of CAP created by dielectric barrier discharge (DBD) and PTS, generated using glow discharge plasma [33], on the pear grafting quality. We also identified the optimal processing parameters: the duration of DBD CAP exposure and the PTS dilution rate.

## 2. Results

### 2.1. Design of the Field Experiment

**Figure 1:**
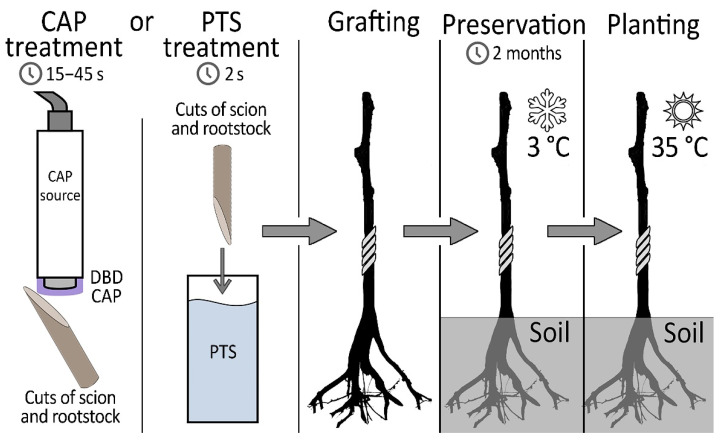
Experimental protocol.

The essence of our proposed method is the treatment of graft and rootstock cuts with DBD CAP or PTS before grafting. Figure 1 shows the sequence of manipulations.

After that, the rootstock–scion combinations were sent for preservation in a refrigerator. After 2 months, planting was carried out in a greenhouse, and further growth of the samples was monitored. The target indicators in determining the quality of the grafting were the root collar diameter, the scion growth, and the resistivity of the grafting zone, which indirectly indicates how well the vascular system was formed in the area of contact between the rootstock and scion. The number of samples in experimental groups and number of surviving samples (1 month after planting in a greenhouse) are presented in Table 1.

### 2.2. Physicochemical Properties of DBD CAP

**Figure 2:**
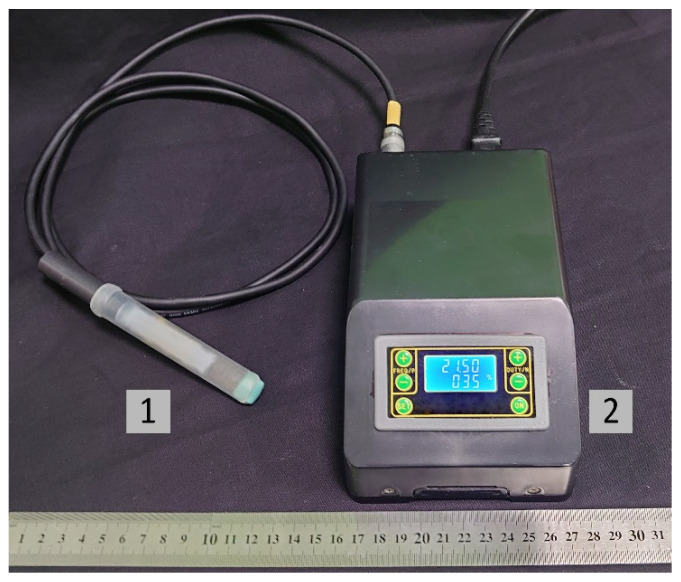
Photo of the “CAPKO-1” device for a CAP generation: 1—an output device with a silicone cap, on the surface of which a DBD CAP is created; 2—power supply unit and control of DBD CAP generation modes. The power consumption of the device is up to 40 W, and input voltage is 220–230 V.

The CAPKO-1 mobile device developed at the GPI RAS (Moscow, Russia) [34] was used as a CAP source for the graft treatment. By changing the output device (Figure 2), this generator can create three types of CAP: plasma jet (with noble gas flow), direct discharge plasma, and dielectric barrier discharge plasma. The principle of the device operation was described in detail previously [31,35,36]. For direct treatment of biological surface, the use of DBD CAP looks promising, since with this type of CAP, it is possible to affect relatively large areas of a temperature-sensitive sample, and at the same time, there are no costs associated with the use of noble gases (for example, helium). Input voltage of the generator is 220–230 V, and power consumption is up to 40 W. The contact surface at the output device is a replaceable cap made of food-grade silicone with a thickness of ~1 mm.

The key inducers of the biological activity of CAP are reactive oxygen (ROS) and nitrogen (RNS) species, for example, H_2_O_2_ and NO_x_^−^, respectively, which appear in the liquid phase (intercellular fluid). The used DBD CAP generator allows one to change the concentration of these compounds in two ways. The first of them is to increase the electric potential at the electrode of the output device or the pulse duration, which leads to an increase in the vibrational and rotational temperatures of gas ions interacting with the intercellular fluid of a biological object. The second way is to increase the time of interaction of the plasma with the target. To identify the optimal treatment of the graft in our work, we used the second approach.

**Figure 3:**
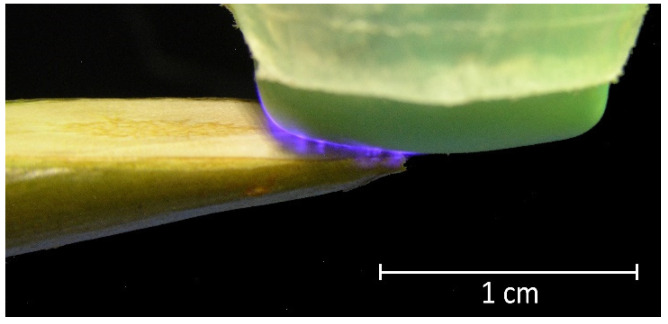
Photo of DBD CAP when processing a pear branch cut. The silicone cap of the output device contacts the surface and moves along the cut during treatment in a multi-pass approach.

**Figure 4:**
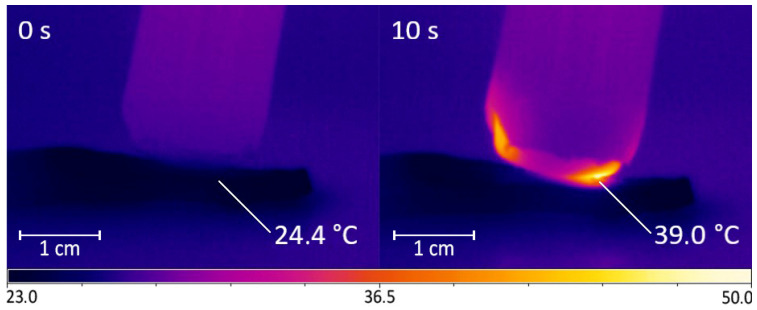
Changing the surface temperature of a pear branch cut during treatment using DBD CAP. During 10 s of exposure, surface temperature rose by (14.6 ± 2.0) °C. Number of repetitions was 5.

The output device of the DBD CAP source was in close contact with the surface of the samples (scion and rootstock). Due to the cylindrical shape of the shoots, an area of no more than 5 × 5 mm was exposed in each moment of time. To process the entire cut and nearby areas, the output device moves across the surface in a multi-pass approach (Figure 3). The multi-pass processing duration was 15, 30, and 45 s, with each surface point being treated for no more than 10 s in total. For 10 s of continuous treatment, the surface heated up by an average of (14.6 ± 2.0) °C (Figure 4); however, with the multi-pass method of surface activation, the maximum temperature was lower, because the surface had time to cool down before re-passing the DBD CAP source. We chose the duration of treatment based on the routine grafting protocol, which requires the fastest connection of scion and rootstock after cutting. We limited the treatment duration to 45 s, as longer exposure can lead to dehydration of the cut.

**Figure 5:**
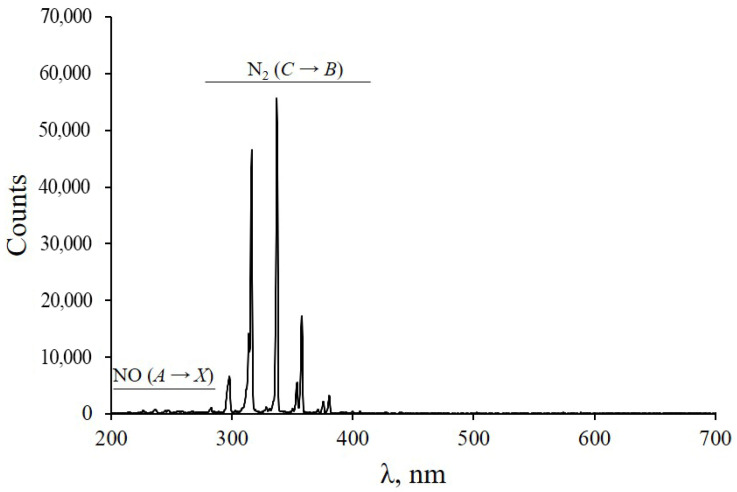
Emission spectrum of DBD CAP in air during branch cut treatment.

Figure 5 shows the emission spectrum of a DBD CAP with a silicone cap taken with an AvaSpec-2048 spectrometer. In the range 200–700 nm, the spectrum consisted of the radiation of the nitrogen N_2_ second positive system (*C*^3^Π*_u_* → *B*^3^Π*_g_*) and NO γ system (*A*^2^Σ^+^ → *X*^2^Π). These emission bands are found in most air discharges.

To assess the production of H_2_O_2_ and NO_2_^−^ ions on the surface of rootstock and scion cuts during processing, we used liquid media as a model object: Milli-Q water and 1% aqueous sucrose solution. According to the degree of RONS generation in deionized water (Milli-Q), different CAP sources can be compared with each other. Sucrose aqueous solution was chosen as the simplest model for xylem sap. This model characterizes RONS generation at the cut surface more closely than the deionized water model.

**Figure 6:**
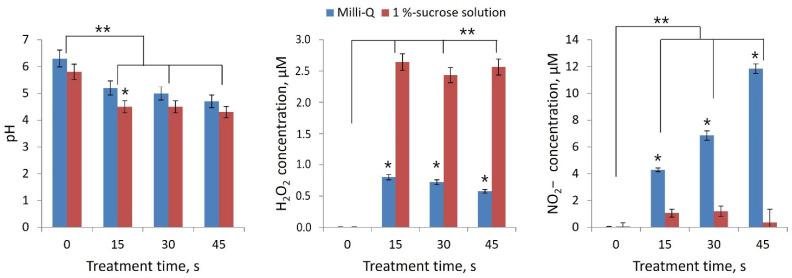
Change in pH and generation of H_2_O_2_ and NO_2_^−^ when DBD CAP was exposed to Milli-Q water and aqueous sucrose solution (1%) for 15, 30, and 45 s. Number of independent experiments was 5. * indicates a significant difference at 5% level in comparison with Milli-Q group with 1%-sucrose solution group at same treatment time (*p* < 0.05, *Mann*–*Whitney U* test). ** indicates a significant difference at 5% level in comparison with the control (treatment time 0 s) (*p* < 0.05, ANOVA). Data are presented as mean values and standard errors of the mean.

The pH when processing Milli-Q water decreased from 6.3 to 4.7, and when processing sucrose solution, from 5.8 to 4.3 (Figure 6). There was a significant difference in the production of H_2_O_2_ and NO_2_^−^ for these two models. After treatment of the sucrose solution for 45 s, an order of magnitude lower concentration of nitrite ion was registered. This is explained by the oxidation of the nitrite ion NO_2_^−^ to nitrate ion NO_3_^−^ when interacting with the ∙OH radical: NO_2_ + ∙OH → HNO_3_ [21,23]. Significantly higher production of ∙OH in the sucrose solution was evidenced by the concentration of hydrogen peroxide H_2_O_2_.

### 2.3. Physicochemical Properties of PTS

The second approach of graft processing that we used in this work is the so-called indirect method. In this case, the biological object is affected by the plasma-treated solution (PTS). To generate the PTS, we used a source of low-temperature plasma, which is formed by high-frequency glow discharge in water vapor. The structure and features of the source operation were described previously [33]. The initial liquid was an aqueous solution of NaCl (0.1 M). The solution was treated with glow discharge for 40 min. The physicochemical properties of the PTS are shown in the Table 2.

Previous experiments [37,38] on planting material processing with this type of PTS have shown that the result can vary over a wide range, up to the death of an object. It is important to find the concentration of PTS that is most effective for a particular biological object and the duration of its exposure. We settled on three options for PTS diluting in deionized water (DW): 1:5, 1:10, and 1:20. When PTS is diluted with deionized water, the concentration of RONS is reduced by the same proportion, and therefore, it allows one to change the degree of exposure of the PTS to the sample. Duration of rootstock and scion cuts immersion in PTS was 2 s. After immersion, the excess liquid was removed by shaking.

### 2.4. Study of the Effectiveness of the Action of DBD CAP and PTS on the Graft

“Lada” cultivar of *Pyrus communis* L. was selected as test sample; it was grafted onto a wild *Pyrus communis* L. The processing of rootstock and scion cuts was carried out in March 2021. For this, DBD CAP (so-called direct treatment) and PTS (so-called indirect treatment) were used. The change in the intensity of treatment, that is, the change in the concentration of the ROS and RNS generated in the surface layer of the object, was carried out during direct treatment by changing the duration of DBD CAP exposure (15, 30, and 45 s), and indirect treatment by PTS diluting in DW in three proportions—1:5 (PTS:DW), 1:10, and 1:20.

**Figure 7:**
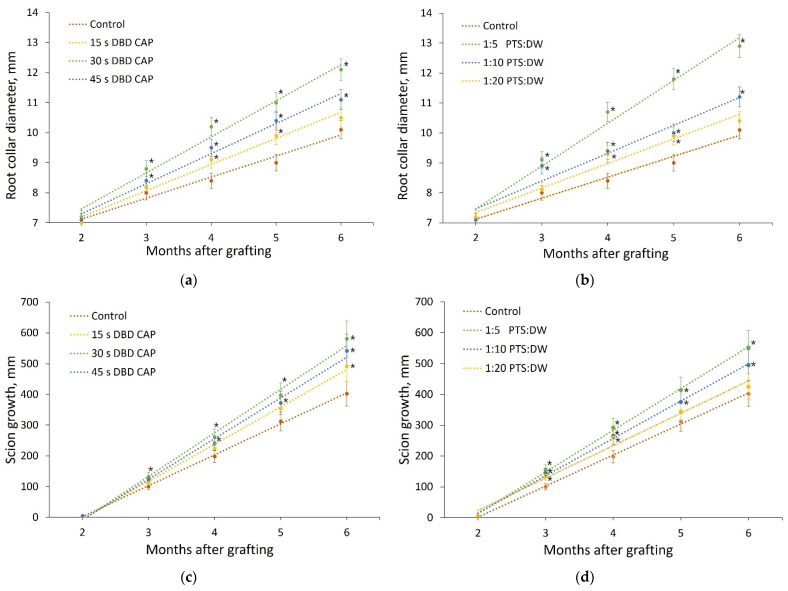
Development of pear shoots within 4 months after planting in a greenhouse at an air temperature of (35 ± 1) °C. The results of the root collar diameter (**a**,**b**) and the scion growth (**c**,**d**) measurements are presented for 7 experimental groups: control, direct treatment with various DBD CAP exposure durations (15 s, 30 s, and 45 s), and indirect treatment with PTS diluted in DW in three proportions (1:5, 1:10, and 1:20). Number of samples in each experimental group is presented in Table 1, and number of measurement repetitions was 5. * indicates a significant difference at 5% level in comparison with the control (*p* < 0.05, ANOVA). Data are presented as mean values and standard errors of the mean.

The state of the samples in each experimental group was monitored for 6 months (until September 2021). For the first 2 months (until May), the shoots were stored in a refrigerator at a temperature of (3 ± 1) °C and a relative air humidity of 80%, and for the next 4 months, the shoots grew in a greenhouse at a temperature of (35 ± 1) °C and an air humidity of 85%. The dynamics of the shoots’ development is shown in Figure 7. The analyzed parameters during the observation were the scion growth and the root collar diameter.

The change in the root collar diameter and the scion growth during 4 months of pear growth in the greenhouse (6 months after the treatment) was well-approximated by a linear function. The values of these parameters after the entire observation period differed significantly in the studied groups.

When treated with DBD CAP for 15 s, the mean root collar diameter was 4% higher than the mean root collar diameter of the controls; for 30 s, it was 20% higher, and for 45 s, it was 10% higher. The scion growth when treating with DBD CAP for 15 s exceeded the scion growth in the control group by 22%; when treated for 30 s, it exceeded control by 44%; and when treated for 45 s—by 35%.

PTS processing showed similar patterns. When diluted 1:20, the average root collar diameter exceeded the average root collar diameter of the control samples by 3%, and the average growth was 6% higher. When diluted 1:10, the average root collar diameter exceeded the average root collar diameter of the control samples by 11%, and the average growth was 23% higher. When diluted 1:5, the average root collar diameter exceeded the average root collar diameter of the control samples by 28%, and the average growth was 37% higher.

**Figure 8:**
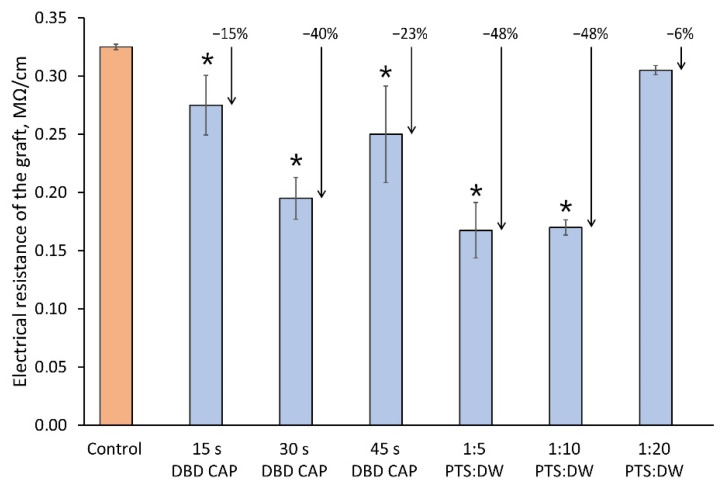
Resistivity of the graft (less is better) that corresponds to vascular system differentiation in the rootstock–scion interface. The measurement approach is described in Section 4.5. Number of samples was 5, and number of measurement repetitions was 5. * indicates a significant difference at 5% level in comparison with the control (*p* < 0.05, ANOVA). Data are presented as mean values and standard errors of the mean.

One of the methods for assessing the vascular system differentiation in the rootstock–scion interface is to measure the electrical conductivity of the graft (less is better) [39]. Comparative results are shown in Figure 8. The lowest value of resistivity compared to the control when processing samples with DBD CAP was at a duration of exposure of 30 s. When processed with PTS, the lowest resistivity values were at 1:5 and 1:10 dilutions.

## 3. Discussion

Recent studies have shown that low-temperature plasma treatment is unique and environmentally friendly. As well as low- and high-voltage electrical discharge [40,41], pulsed magnetic field [42], and UV-A rays [43], CAP technology has shown many advantages in the agriculture sector [44,45]. Deferent plasma generation methods and setups have been presented and have also shown that plasma-treated solutions can be used along with CAP sources for “indirect” treatment [46,47]. The influence of plasma treatment on DNA damage, gene expression, enzymatic activity, morphological and chemical changes, germination, and resistance to stress is under research [48,49,50]. Many papers have reported promising results in this wide variety of applications [51,52].

For example, dielectric barrier discharge cold atmospheric plasma could significantly improve basil *(Ocimum basilicum* L. cv. Genovese Gigante) plants’ physiological and biochemical traits, including ion leakage, water relative content, proline and protein accumulation, chlorophyll and carotenoid contents, and antioxidant activity [53]. High-voltage electrical discharge plasma technology showed the potential to improve drought and salt tolerance in wheat [40]. Short-time pre-sowing treatment of stevia seeds with CAP and electro-magnetic field could enhance of biosynthesis of steviol glycosides responsible for the sweetness [54]. A positive effect of CAP on soybean germination could be achieved, and the percentage of germination increased by almost 20% compared to the untreated control [55]. Additionally, CAP treatment may be applicable in postharvest and food production as it reduces the frequency and diversity of fungal strains [56].

Despite numerous studies in the field of plasma agriculture, the use of CAP for plants grafting is not covered in the studies. For optimal plant growth after grafting, the formation of a water and nutrient transport system and the quality of the rootstock–scion assemblage is of fundamental importance. As we showed in this paper, the use of DBD CAP and PTS allows one to modify existing grafting techniques to improve a number of key performance indicators and seems promising to boost productivity.

Both parts of the rootstock–scion combination were treated with either DBD CAP or PTS. DBD CAP treatment was carried out using a scanning multi-pass approach for 15, 30, or 45 s. Moreover, each local area of the cut was not exposed to DBD CAP for more than 10 s in total, in order to minimize heating of the samples. The discharge was activated between a covered dielectric driven electrode and a specimen surface applied with a floating (free) potential. The generation of RONS in liquids upon exposure to DBD CAP is shown in the Figure 6. In the xylem sap model, a significant amount of hydrogen peroxide was produced, which plays an important role in a number of processes for plants [57].

The concentration of RONS in the generated PTS is shown in Table 2. The reduction in concentration was achieved by diluting PTS in DW (Milli-Q); in this case, the concentration of RONS was changed in the same proportion. This approach made it possible to choose the optimal PTS composition without changing the power characteristics of the plasma generator and the treatment duration. PTS was diluted in DW in three proportions: 1:5, 1:10, 1:20. The scion and rootstock were immersed in the PTS solution for 2 s.

After treatment, grafting was carried out immediately using the «whip and tongue» technique. The combined effect of DBD CAP and PTS has not been evaluated, and therefore, this is a prospect for future research.

From the cumulative results of the measurements, the most effective treatment modes with the DBD CAP were 30 and 45 s of exposure. At the same time, treatment for 45 s showed a slight decrease in the quality of the graft. We associated this with cuts drying due to a longer exposure to the air during all grafting stages. Submerging the cuts in the PTS also resulted in a significant improvement in the key performance indicators of the graft. The most suitable dilutions were in the ratio of 1:5 and 1:10; however, during this series of experiments, we did not reveal the maximum allowable concentration of PTS, the achievement of which leads to growth inhibition. Additional attention will be paid to solving this problem in future experiments.

The results can be explained by the action of several factors.

1. When CAP is applied to wood surfaces, a significant reduction in surface roughness can be achieved [58]. This helps to reduce the total volume of air gaps between the grafted parts, and consequently, to reduce the square of the insulating layer, which is formed from the contents of damaged cells and slows down the graft union formation [59].
2. Surface activation occurs. The activation process is a multi-stage modification of the uppermost layer and near-surface region up to 300 µm in depth [32] by plasma components: electric field, ultraviolet light, electrons, and reactive nitrogen and oxygen species. In particular, metastable nitrogen and ultraviolet photons from the NO-γ system, which are generated in the DBD CAP (Figure 5), interact with ambient oxygen to form ozone and atomic oxygen. This contributes to the lignification of the contact zone [60], which is necessary for the formation of a new vascular system [39].
3. Surface activation leads to an increase in the O/C ratio on the surface [61], that is, an increase in polar oxygen-containing functional groups: CO, OC=O, ^−^OH, etc. [62,63]. This contributes to a significant improvement in surface wettability.

Taken together, these effects significantly improve the adhesive properties of the rootstock and scion surfaces and the resistance of the grafted plant to subsequent physical stresses.

PTS processing from the entire set of operating factors is limited by the action of long-lived ROS and RNS. Nevertheless, as shown during the experiment, the selection of the optimal concentration of PTS could achieve the same efficacy as with the treatment with DBD CAP.

The physiological responses of plants to the plasma treatment may be variety-dependent due to different genetic profiles and other abiotic factors [64], and the complex mechanisms of CAP interactions with biological objects are not fully understood. Thus, there are great prospects in the study of the use of CAP and PTS for plant grafting.

## 4. Materials and Methods

### 4.1. CAP Generation Method

**Figure 9:**
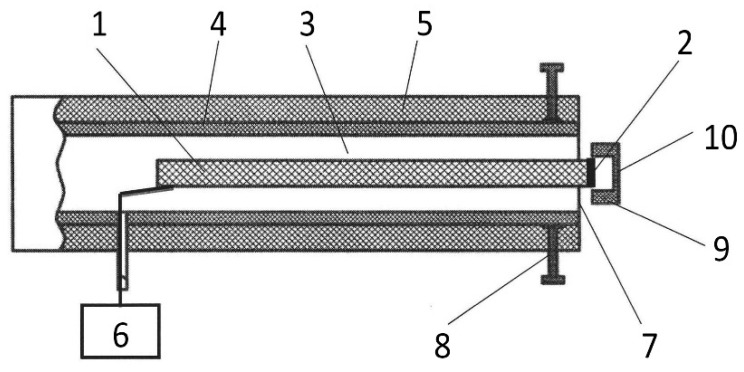
Scheme of the CAP generator output device: 1—piezotransformer, 2—discharge electrode, 3—ionization chamber, 4—dielectric tube, 5—hard case, 6—voltage generator, 7—output end of the dielectric tube, 8—device for changing the shape of the output end of the tube, 9—dielectric cap, and 10—dielectric layer.

For the processing of the stock and scion cuts, a cold plasma source “CAPKO-1” developed by our team was used [34]. The operating principle and characteristics are described in detail in [31], and the appearance is shown in Figure 2. The generator was set to create a DBD CAP. The output device of the generator (Figure 9) is a dielectric tube (4) fixed in a hard case (5), forming an ionization chamber (3), inside which a piezotransformer (PT) (1) is installed, so as not to impede the mechanical vibrations that occur during the PT operation. A low-voltage alternating current of a resonant frequency (60 V, 21.5 kHz) from a generator (6) is supplied to the input part of the PT. A high-voltage of ~6 kV appears at the discharge electrode (2), which is used to create a plasma. This design of the CAP generator contains a cap (9) made of food-grade silicone 1 mm thick (10), tightly fitting the PT’s output, which allows it to operate in a dielectric barrier discharge mode at ~1 mm from the cap surface.

The emission spectra of DBD CAP presented in Figure 5 were recorded using an AvaSpec-2048 spectrometer (Avantes, Apeldoorn, The Netherlands). The change in object temperature during processing (Figure 4) was determined using an Optris PI 640 infrared camera (Optris, GmbH, Berlin, Germany) and Optris PIX Connect software (Optris, GmbH, Berlin, Germany).

### 4.2. PTS Generation Method

**Figure 10:**
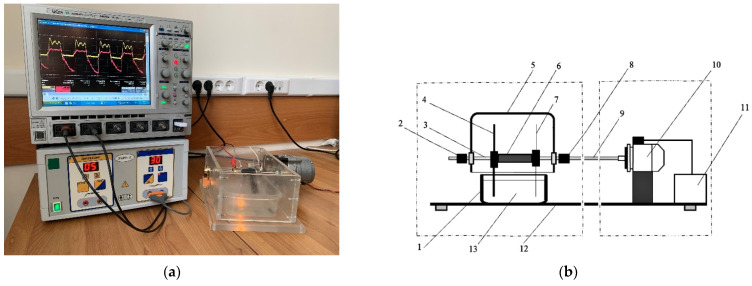
The photo (**a**) and the structure (**b**) of the PTS generator: 1—tank with activated solution; 2—neutral electrode (NE) brush; 3—rotor axis; 4—replaceable parts of the neutral electrode; 5—reactor lid; 6—dielectric loading; 7—replaceable active electrodes; 8—active electrode (AE) brush; 9—kinematic axis; 10—electric motor; 11—controlled power source; 12—platform; and 13—aqueous solution.

The PTS was created using a glow discharge plasma [33]. The device consists of a high-frequency current generator, a plasma-chemical reactor, and a rotor (Figure 10). A weak aqueous solution of a strong electrolyte is poured into container 1. Active (7) and neutral (4) electrodes are immersed in an aqueous solution (13). High-frequency current is supplied to the electrodes through brushes (2) and (8) located on the rotor axis (3). Simultaneously with the high-frequency current, the rotor unit B is turned on. The electrodes are rotated by an electric motor (10), which is powered from a controlled source (11).

A current with a frequency of 440 kHz and a shape close to sinusoidal is supplied to the electrodes. The maximum output power of the power supply is 450 W. The initial liquid was an aqueous solution of NaCl (0.1 M). The solution was treated with a glow discharge plasma for 40 min.

### 4.3. Physicochemical Properties of Aqueous Solutions

The content of nitrite and nitrate anions in the samples was determined using the Griss reagent according to the method described previously [37,65] using a Multiscan FC plate reader (TermoScintific, Vaanta, Finland), and the optical density of the medium was measured at a wavelength of 546 nm. Sodium nitrite and sodium nitrate solutions of known concentration were used for calibration. Number of repetitions was 5.

Redox potential, pH, and electrical conductivity were measured on an S470 SevenExcellence high-precision measuring station (Mettler Toledo, Columbus, OH, USA). The sensor electrodes InLab Expert Pro-ISM and InLab731-ISM (Mettler Toledo) were used. During measurements, aqueous solutions were mixed in a laminar mode using a magnetic stirrer (rotation frequency 3 Hz). All measurements were carried out at a solution temperature of 20 ± 1 °C. Number of repetitions was 5. The experimental measurement details were described previously [66].

The concentration of molecular oxygen dissolved in water solutions was measured using an AKPM-1-02 polarograph (Bioanalytical systems and sensors, Moscow, Russia) [67]. The measurements took into account the atmospheric pressure, measured with a PRX-7001t (Casio, Tokyo, Japan), and the temperature of the samples, measured with a thermocompensating electrode. All measurements were carried out at a solution temperature of 20 ± 1 °C. Number of repetitions was 5. The experimental measurement details were described previously [68].

For the quantitative determination of hydrogen peroxide in aqueous solutions, a highly sensitive method of enhanced chemiluminescence in the luminol-p-iodophenol-horseradish peroxidase system was used [69,70,71]. The luminescence intensity was determined using a Biotox-7A chemiluminometer (ANO ICE, Moscow, Russia). The initial concentration of hydrogen peroxide used for calibration was determined spectrophotometrically at a wavelength of 240 nm with a molar absorption coefficient of 43.6 (M−1 × cm^−1^). The “counting solution” contained: 1 cM Tris-HCl buffer pH 8.5, 50 µM p-iodophenol, 50 µM luminol, and 10 nM horseradish peroxidase. Number of repetitions was 5.

### 4.4. Plants Samples and Field Experiment

**Figure 11:**
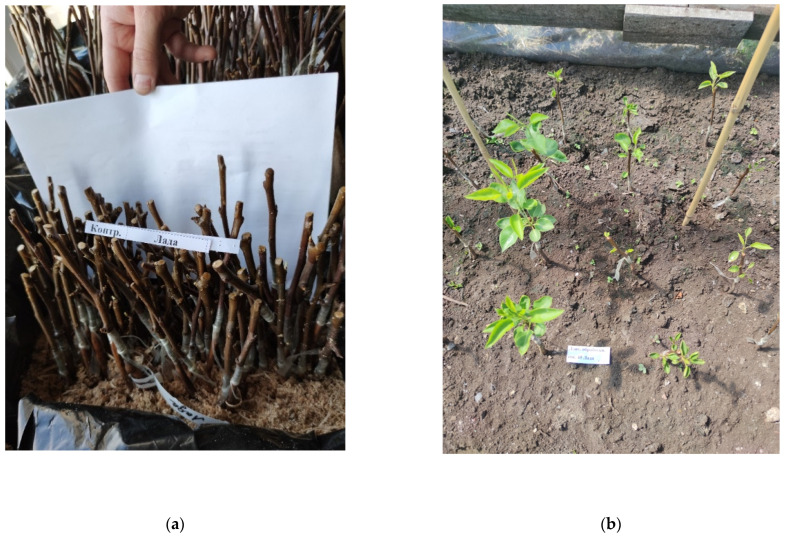
Treated and grafted pear samples of the “Lada” cultivar: (**a**) before being sent for preservation (2 months) in a refrigerator (3 ± 1) °C; (**b**) 1 month after planting in a greenhouse (35 ± 1) °C.

The treatment of samples and their planting were carried out in March 2021 in the nursery of Institute for Engineering and Environmental Problems in Agricultural Production—branch of “Federal Scientific Agroengineering Center VIM” (Saint Petersburg, Russia). The selection of samples for the rootstock and scion was carried out in accordance with the requirements for the quality of fruit crops GOST R 53135-2008. “A wild pear *Pyrus communis* L. was used as a rootstock because it is weather resistant, and “Lada” cultivar (#8007810 in the registry of FSBI «Gossortcommission») of *Pyrus communis* L. was used as a scion because this cultivar is the most demanded and significant for the Russian region. In each experimental group, including the control (without any treatment), 10 samples were prepared. After treatment, the grafted shoots were sent for preservation in a refrigerator with an air temperature of (3 ± 1) °C, relative air humidity of 80%, and humidity in the root zone of 90%. After 2 months, the samples were removed from the refrigerator and randomly planted in a greenhouse with an air temperature of (35 ± 1) °C and an air humidity of 85% (Figure 11). The scion growth and the diameter of the root collar were measured monthly. Observations were carried out within 4 months after planting.

### 4.5. Graft Conductivity Measurements

**Figure 12:**
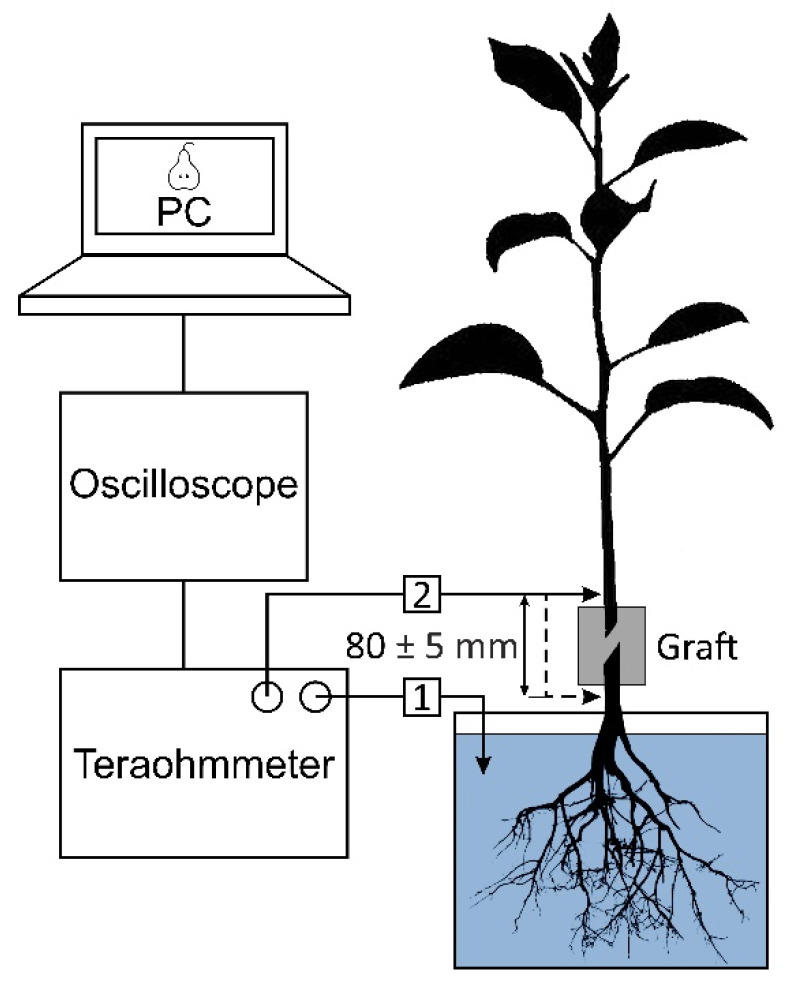
Measurement of the electrical resistance of the graft in a pear seedling 6 months after treatment. The seedling was immersed in the stock solution, and the measuring electrodes were made of silver and coated with a layer of silver chloride. 1—electrode immersed in stock solution; 2—electrode inserted into the cambian layer of the bark. The resistance of the graft was determined by calculating the difference in resistance between two points: when (2) located above the graft and located below it. Number of samples in each group was 5, and number of measurement repetitions was 5.

To assess the quality of rootstock–scion formation, measurements of the electrical resistance (impedance) of the cambium layer in the graft were carried out. After 4 months of growth of seedlings in the greenhouse (after 6 months from the moment of processing), they were delivered to the laboratory. The root system of the seedlings was washed in running water and placed in stock solution: KNO_3_ (5 mM), Ca(NO_3_)_2_∙4H_2_O (2.5 mM), MgSO_4_∙7H_2_O (2 mM), and NH_4_NO_3_ (1 mM). The resistance was measured using an E6-13A teraohmmeter (Radio Factory RET, Tallinn, Estonia) and needle-type electrodes with a diameter of 0.7 mm, made of a silver wire coated with silver chloride. One probe was immersed in the stock solution, and the second was introduced into the cambian layer of the bark (Figure 12). The resistance of the graft was determined by calculating the difference in resistance between two points: located above the graft and located below it. The distance between these points was 80 ± 5 mm. To minimize the error, measurements were repeated five times. An AKIP-4122/1 digital oscilloscope (Prist, Moscow, Russia) was connected to the teraohmmeter. This oscilloscope was connected to a personal computer with the PicoDiagnostics software (Pico Technology, Cambridgeshire, UK) installed.

### 4.6. Statistics

Data are presented as means ± SEM. The normality of distributions was established by the Kolmogorov-Smirnov criterion. When the distribution was normal, Student’s *t*-test was used to compare independent groups. When the distribution differed from normal, the Mann–Whitney *U* test was used to compare two independent groups. ANOVA was used for multiple comparisons.

## 5. Conclusions

For the first time, an approach using DBD CAP and PTS was proposed and described, which allows one to improve existing methods of garden plants grafting and significantly increase the rate of plant entry into the market. The test sample was a *Pyrus communis* L. of the “Lada” cultivar.

The modes of DBD CAP generation, treatment duration, and PTS concentration are described, which makes it possible to achieve significantly more attractive key indicators of the graft quality compared to control samples.

1. The treatment of the cuts surface using the plasma source “CAPKO-1” for 30–45 s increased the scion growth by 35–44% and the root collar diameter by 10–20%. In this case, the electrical resistance of the graft union, which characterizes the differentiation of the functional vascular system (the less, the better), decreased by 20–40%.
2. Cut surface treatment with the PTS described in this article (aqueous solution of NaCl treated for 40 min with a glow discharge) may require preliminary dilution in DW. The result obtained for a 1:5 ratio demonstrated an increase in the scion growth by 37% compared to the control and an increase in the root collar diameter by 28%. The electrical resistance of the graft was reduced by 48%.

The results demonstrated that use of CAP and PTS in plant grafting seems promising to boost productivity. However, the optimum duration of exposure to DBD CAP and the optimum dilution of PTS must be specified for each rootstock–scion combination. Furthermore, complex physical and chemical processes during CAP interaction and after it can be investigated in detail.

## Figures and Tables

**Table 1:** Number of samples in experimental groups and number of surviving samples 1 month after planting in a greenhouse.

	Control	Direct Treatment			Indirect Treatment		
Processing duration, seconds	0	15	30	45	2	2	2
Proportion of PTS diluted in deionized water	−	−	−	−	1:5	1:10	1:20
Number of samples	10	10	10	10	10	10	10
Number of surviving samples (1 month after planting in a greenhouse)	8	9	10	9	10	10	9

**Table 2:** Physicochemical properties of PTS after treatment with high-frequency glow discharge of an aqueous solution of NaCl 0.1M.

Exposure Time, min	Electrical Conductivity, mS/cm	O_2_, µM	pH	Redox, mV	NO_3_^−^, mM	H_2_O_2_, mM
0	7.3 ± 0.5	273 ± 5	6.7 ± 0.1	303 ± 7	<0.01	<0.01
40	24.9 ± 1.2 *	261 ± 8	8.3 ± 0.2 *	598 ± 26 *	22.05 ± 0.98 *	7.12 ± 0.68 *

* Statistical differences relative to control (*p* < 0.05, Student’s *t*-test).
